# Supplementary material for: Identification and Functional Analysis of KH Family Genes Associated with Salt Stress in Rice
Source: Int J Mol Sci. 2024 May 29;25(11):5950. doi: 10.3390/ijms25115950 (PMC11172612; doi:10.3390/ijms25115950)
Supplement: Supplementary file 1 [file ijms-25-05950-s001.zip › ijms-2944043-supplementary.pdf]

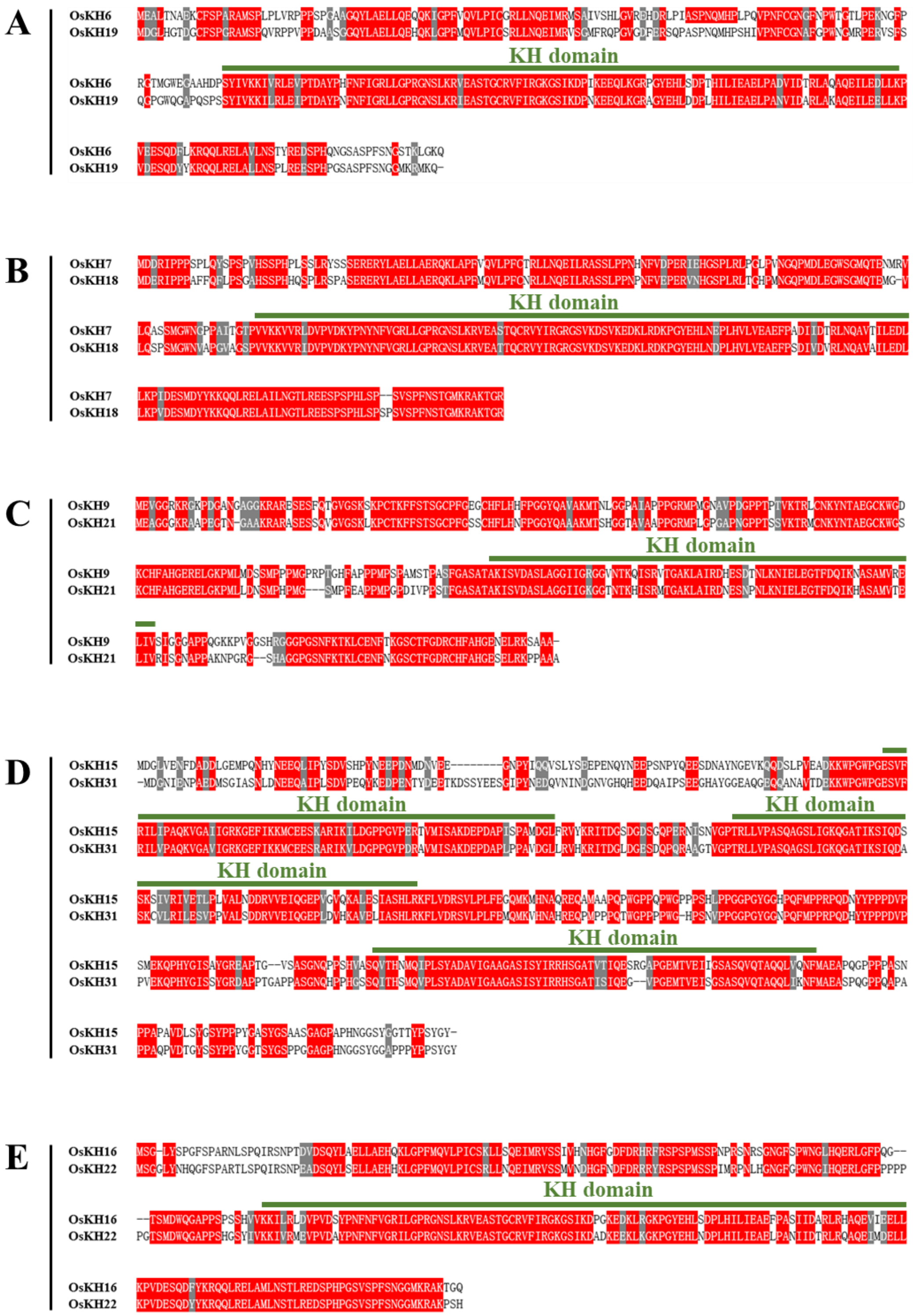

Figure S1. Alignment of five pairs of KH protein sequences with collinearity in rice

The red part represents the part on the exact alignment, and the areas marked in green are KH domains.

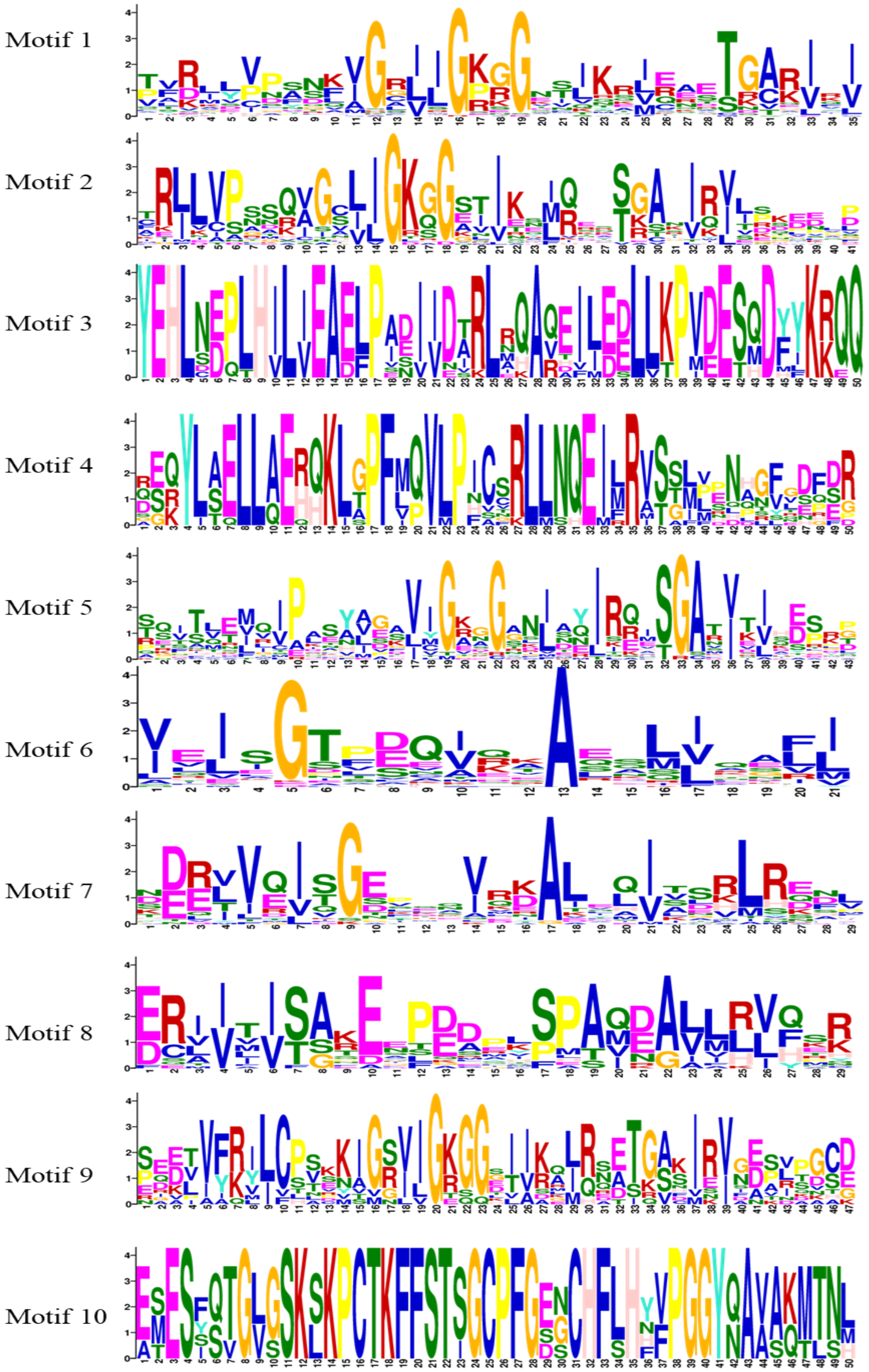

Figure S2. The amino acid sequence of each conserved motif of KH proteins

The amino acid sequence of each conserved motif contained in KH proteins, and each amino acid was present in a different color.

Table S1. Primers used for qRT-PCR of KH family genes in rice

Gene	Forward primer (5'-3')	Reverse primer (5'-3')
OsKH1	ATCCACCCAAGCCTTGTTGA	AGGGCTCCTTTCTTGCTTCT
OsKH2	GGGAATGTCATTGGCAAGGG	GCCATCAACAGAACAAGCCA
OsKH3	TGACGCCCAAGATTCTCACT	ATCTTGATCCTGGCACCACT
OsKH4	TTGTCAGCCTCCTTGCTGAT	GAAGCGGACATCATCTGTGG
OsKH5	AACCTGAAGGCAAGAAGGGT	TGCGATGTCAAATCCAAGCA
OsKH6	ACAGCAGAAGATTGGTCCCT	TGGTTTGGACTTGCAATGGG
OsKH7	AGTTCTTCCCTTCTGCACCA	GGAGGCCAGGTAACCTCAAT
OsKH8	CCCTCAAAGTTCTCCATGCC	ATGTGCCTCACCTACTGCTT
OsKH9	TGCCAATGGGAAATGCTGTT	TTGCATCCCTCTGCAGTGTT
OsKH10	CAGCCTCCTGCATATGCTTC	TCCAGTCTGCTGAGAGTAGC
OsKH11	TCTCAAGGCAGTTCCTCTGG	GTTGCTTCCACTCGCTTCAA
OsKH12	GACCATCAGATGCGTGCAAT	GGAAACCAAGGCCTGCATAG
OsKH13	TGTGCAGACTTCGTACATGC	ACGTGACAGATGCTCTCCTC
OsKH14	AGTTACCGTTCGGTTGCTTG	CATCACCACTGATGGCACAC
OsKH15	CTGCTAGTACCAGCTTCCCA	TGGCTATCGATTCCAGTGCT
OsKH16	TGCGGGTATCAAGCATTGTC	TCTTGGTGTAGCCCATTCCA
OsKH17	AGCACTATCGGATGCCCTTT	ACAGCTGGGTTGCCTAGATT
OsKH18	TTGAGGGCATCTTCTTTGCC	TTGCATTCCTGACCATCCCT
OsKH19	ATCGTGCCCAATTTCTGTGG	CGGTATTTCCAACCGCAAGA
OsKH20	CATTGCTGCACCTCCAAACT	TTGCACTGGTCTGTCTCTGT
OsKH21	TCGTCTCAAGTGGGTGTAGG	AGGAGCACCAGGTCCTAAAG
OsKH22	AGTCCGAGTCCCATGTCTTC	AGGAGGAGGAGGAAAGCCTA
OsKH24	TGCTCCACCAATTCCTTCCT	AGGTGGTGGTCCTGTGTATG
OsKH25	GGTGGCGGTTACGACTACTA	CAGACTGCGGATACTGAGGT
OsKH26	GGCTGTCTTATTGGCAAGGG	GCTCATCACTGGAAGATGCC
OsKH27	CTCCAGCTCAGGATGCAGTA	TTCGGATACCAGCTCCTGTT
OsKH29	AGGCTCCACCACAATTACCA	ATGATACTGCAGTGGCCTGT
OsKH30	ACTCAAGATCGCAGCACTGA	GGTTCCCGTTCGAGAAACAG
OsKH31	ATGCACCATTACCTCCTGCT	TTGAGAAGCTGGCACCAGTA
UBQ	ACCACTTCGACCGCCACTACT	ACGCCTAAGCCTGCTGGTT

Table S2. Primers used for qRT-PCR of salt-responsive marker genes in rice

Gene	Forward primer (5'-3')	Reverse primer (5'-3')
LEA3	TGAAGAGCACGGTGGTCGG	GGCAGAGGTGTCCTTGTTGG
DREB1A	GACGTCCTGAGTGACATG	GTAGCTCCAGAGTGGGAC
SKC1	CATCGTCGTCGAGGTTATCA	TCCCTTGTTTGCTCCACTTC
HKT2;1	CGGCGTTTCTGGCATCA	GCACCGAACAATGTGACCAA
AKT1	CAGTAATGAATGGGATGCAGAG	CCCCTTTCTTGGAAATCAACAG
iSAP8	CATGAACATGTGCTCAAAG	GACAATGCTGTCGATAGAG
NHX2	TCGTCGCGATCAACATCTTCGT	TCCCACCACTGACGAGCAGA
SOS2	TCGCCATGAAGGTGCTCGAC	TCGCCTCCAGTGATAAGCTCCA
DSR2	GAACTACCGCCAAAAGATCG	CGTTGTAAGAGGATGACCG
